# Effects of Dining-focused Life Enhancement Program in Welfare Facilities for Seniors in Japan

**DOI:** 10.31372/20200502.1089

**Published:** 2020

**Authors:** Reiko Sakashita, Hiroshi Ono, Takuichi Sato, Miho Takami, Woesook Kim, Eiko Nakanishi, Hiroyuki Kusumoto, Masayo Hamasaki, Misao Hamada

**Affiliations:** a University of Hyogo, Japan; b Niigata University Graduate School of Health Sciences, Japan; c Special Nursing Home for the Aged Altenheim, Japan; d Medical Cooperation, Mikiyu, Japan; e Social Welfare Corporation Lavita, Japan

**Keywords:** dining, elderly, food intake, life enhancement, oral care, welfare facility

## Abstract

This study evaluated the effectiveness of a life-enhancement program designed to focus on dining conditions in welfare facilities for seniors living in Japan. Effectiveness was specifically evaluated based on whether improvements were achieved in (1) nutritional status, (2) oral health, (3) frequency of fever, and (4) vitality of appetite across three sites. As part of a comprehensive-care initiative that began with dining support, the program consisted of two main components: (1) a 3-month intensive program comprised of (a) collective experiential learning for residents and staff (including nutritionists, nurses, and physiotherapists) and (b) a tailor-made individual program for residents followed by (2) a 3-month continuation program. Participants included 168 individuals (31 males and 137 females) from a total of three facilities (average age was 85.9 [60–104] years). Results showed that the intensive program significantly improved nutritional status (*e.g.*, BMI, caloric intake, and water intake; *P* < 0.000–0.005) and tongue movement (*P* < 0.000) while significantly reducing dental-plaque and tongue-coating indices (*P* < 0.000). Significant improvements were also achieved for degree of appetite and vitality indices (*P* < 0.000–0.001). However, incidences of fever were not reduced. These findings indicate that the program effectively improved nutritional status, oral health, vitality, and appetite. However, these effects did not sufficiently remain once the program was finished, thus suggesting the need for a continuous intervention.

## Introduction

Elderly malnutrition is currently a serious issue. In Japan, the pooled prevalence rates of frailty have been measured at 20.4% and 35.1% for those aged 80–84 and ≥85, respectively (Kojima et al., 2017). In this context, the major causes of malnutrition include advanced age, apathy/depression, disease (*e.g.*, cancer, diabetes, cardiac issues, and gastrointestinal problems), the inability to purchase, cook, and/or consume food, the inability to chew and/or swallow, limited mobility, sensory loss, treatment requirements (*e.g.*, ventilation, surgery, and draining tubes), and drug therapies (Kubrack & Jensen, 2007). These issues are especially important because malnutrition is known to increase the probabilities and rates of several other conditions, including infections, pressure ulcers, the length of hospital stay, and duration of convalescence after acute illness, and mortality (Agarwal, Miller, Yaxley, & Isenring, 2013). Further, research has shown that mortality rates steadily increase as BMI levels decrease (Winter, MacInnis, Wattanapenpaiboon, & Nowson, 2014). Therefore, the Japanese Ministry of Health, Labour and Welfare (JMHLW) has identified “nutritional improvement” as a pillar strategy to prevent the need for long-term care (JMHLW, 2012). The Ministry aims to improve elderly people’s nutritional status through dining, which is a vital daily activity, and thereby promote their physical condition, communication, and social participation and improve their daily living functions (JMHLW, 2012). Dining provides broader benefits to physical health, psychological and social well-being, life enrichment, and happiness (Curle & Keller, 2010; Morley, 2013). However, reports indicate that many elderly persons who live in welfare facilities are suffering from poor nutrition (Hirose et al., 2014; Salva et al., 2009). As increasing numbers of elderly residents struggle with health problems from poor nutrition, the type of care that focuses not only on their illnesses and impediments but also on their quality of life (QOL) is becoming increasingly important.

Dining is a complex activity. This study’s authors previously conducted a conceptual analysis in which dining was defined as a fundamental behavior in daily life for intake of nutrients, tasting and savoring, and developing personal relationships, but that sometime threatens life by choking and aspiration (Sakashita et al., 2016). The analysis also identified the following conditions as antecedent factors for dining: need of nutrients, functioning organs, awaking, self-esteem, appetite/desire to eat, existence of proper food, comfortable environment, persons to meal with, and wishes by family (Sakashita et al., 2016). These factors must be comprehensively satisfied in order to provide appropriate dining support for the elderly. It is therefore essential to construct a care plan that involves multiple such perspectives. However, services provided in the healthcare setting tend to emphasize issues of physical assistance, such as those targeting eating and swallowing disorders (Martha, 1970). In this context, there is a general lack of consideration for the highly important aspects of elderly dining assistance that addresses individual personalities and preferences.

In this study, we developed and implemented a comprehensive life-enhancement program that focused on dining conditions (Dining-focused Life Enhancement Program) in welfare facilities for seniors in Japan. The hypothesis is that improving oral condition and eating environment will promote appetite to increase nutritional intake from vitalization and reduced fever incidents.

We then examined its effects over the course of a (1) 3-month intensive intervention and (2) subsequent 3-month continuation program. Finally, we measured the interventional effects 6 months after the end of the continuation program (i.e., 12 months after beginning the first intervention).

### Study Purpose and Research Questions

This study clarified the effects of a dining-focused life-enhancement program based on the four following research questions:

- Did the program improve nutritional status among participants?
- Did the program improve oral health among participants?
- Did the program improve vitality and appetite among participants?
- Did the program reduce the frequency of fever among participants?

## Methods

### Study Design and Period

This was a quasi-experimental before and after study. It was conducted between February 2014 and March 2015.

### Dining-focused Life Enhancement Program

**Figure 1.**
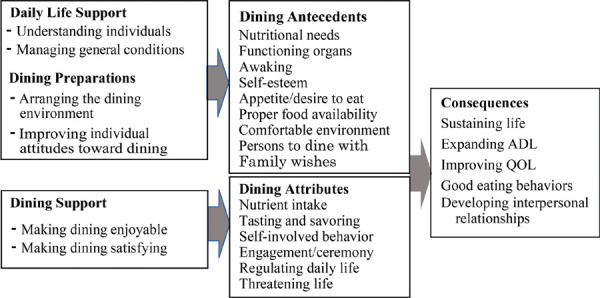
Care model for a life-enhancement program focused on dining conditions at welfare facilities for seniors.

We developed a Dining-focused Life Enhancement Program based on our previous conceptual analysis of the dining experience (Sakashita et al., 2016). Figure 1 shows the care model based on the analysis.

**Figure 2.**
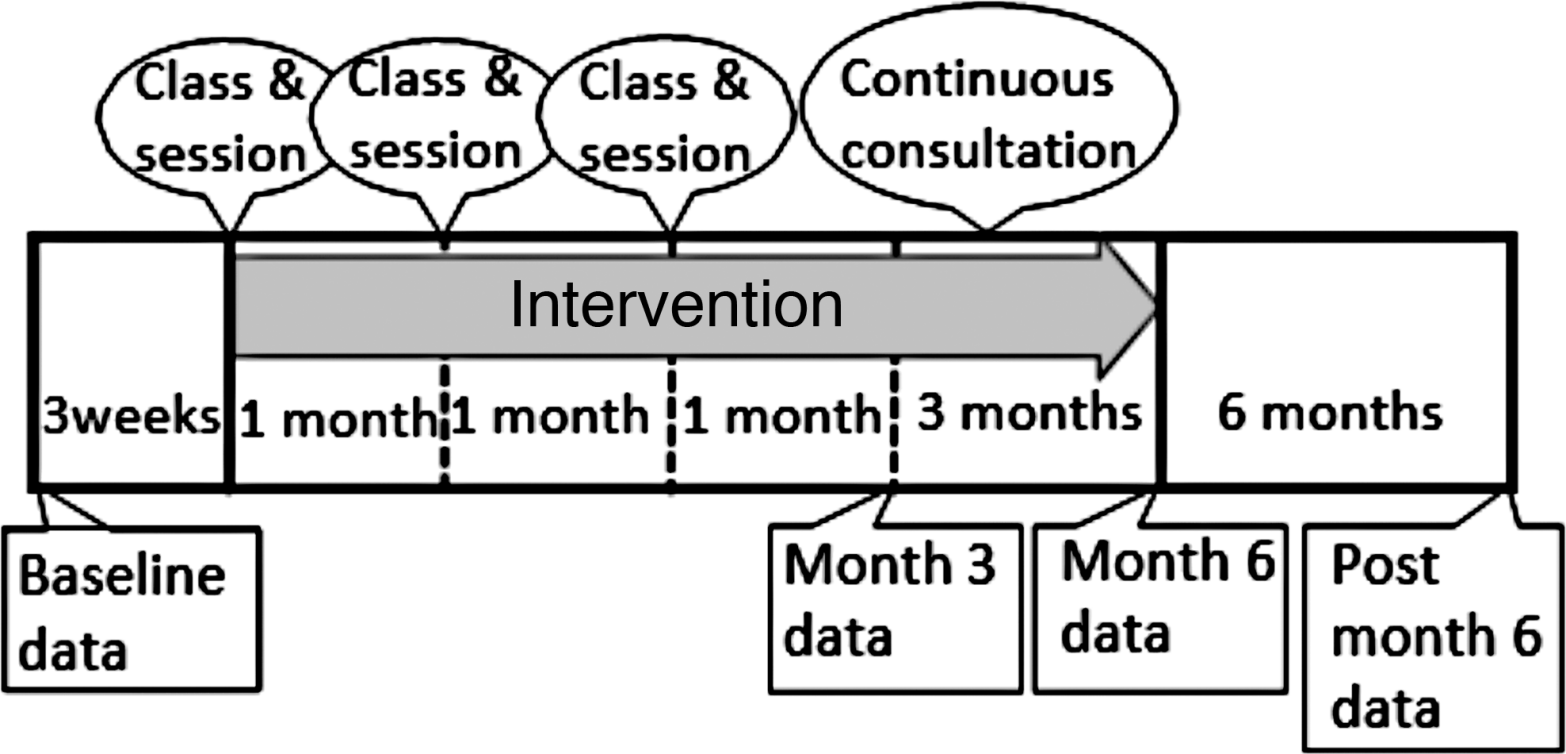
Research schedule.

The program was developed following a pilot study (Sakashita et al., 2016), which suggested that this program was effective in improving nutritional status, but further study was needed with a larger sample size at multiple sites. The program was implemented according to a 6-month intervention program consisting of two main parts: (1) a 3-month intensive program designed to promote Daily Life Support, Dining Preparations, and Dining Support in Figure 1 and (2) a 3-month continuation program designed to maintain and further promote those improvements (Figure 2). The data were collected 6 months after the program ended to evaluate continuity of the program effect. As the program was simultaneously implemented at three separate facilities for the elderly, we determined its contents based on a draft program created by an expert team comprised of nurses, doctors, dentists, dental hygienists, psychologists, physical therapists, and experts in social welfare while incorporating the opinions of facility staff members including dietitians, care workers, nurses, and physical therapists.

### Part 1: The 3-Month Intensive Program

#### Collective experience learnings

Collective experiential learning sessions were held to help participants improve their knowledge levels and skills of Dining Preparations and Dining Support while stimulating the motivation to improve and promote the continuation of improvement activities by providing opportunities for mutual support. Group learning sessions were separately held for residents and staff members (i.e., the nutritionists, nurses, and physiotherapists). Each group completed one session per month during the 3-month intensive intervention period.

#### Learning sessions for residents

Resident learning sessions lasted approximately 30 minutes each, which were designed to increase their desire to eat and appetite rather than improving their ability to do so. At each session, we divided residents into small groups of four or five and assigned one or two nurses/staff members to each group, while an expert in dementia nursing worked as a session facilitator. During the first session, residents discussed memorable food items and confirmed what tastes they most enjoyed while looking at photographs and taking drinks (*e.g.*, green tea, sweet red-bean soup, juice, and some carbonated drink). Residents then considered what constituted a desirable menu. Following this, a discussion was held with families and staff members to help fulfill these wishes. During the second session, residents participated in various activities designed to improve their oral function, thus enabling them to eat items from their desired menus. Specific activities included oral care, swallowing exercises, and therapeutic recreation (*e.g.*, singing). During the third session, residents participated in games designed to promote correct eating posture and movement. This further promoted the ability to eat food items from their desired menus.

#### Learning sessions for staff members

Learning sessions were also provided to staff members who delivered care to residents, including care workers, nurses, and physical therapists. The sessions were specifically designed to help staff members understand the individuality of residents based on mutual trust and to improve the individual’s attitude toward dining. The first session focused on care skills for residents with declining cognitive function. This included a practice exercise in which staff members attempted to discern nonverbal cues and reactions, followed by a feedback session and exercise for assessing eating functions, swallowing functions, and oral-care techniques. The second session helped staff members review their care practices and asked them to find ways to encourage dining experiences based on the caregiver–resident relationship. Finally, the third session prompted staff members to discuss cases in which improvements were made to dining conditions during the previous 2 months of intervention. They also shared information about care practices that effectively improved dining experiences for residents.

#### Tailor-made programs for each client

The standard intervention protocol was used in each of the facilities. We conducted a preliminary survey among participants 3 weeks prior to the intervention. This included the welfare facility residents, their families, and staff members. The purpose was to investigate what foods residents desired while assessing food intake and oral-health status for arranging dining antecedents in Figure 1. The researchers gained extensive information about residents’ dietary status, which was used to develop tailor-made programs for each person once per month (three total times). Every resident had their program modified with one to three related items from the following list, depending on their condition:

- Devising ways to allow the resident to savor and enjoy food
- Reviewing the consistency of foods eaten by the resident
- Oral hygiene (oral cleaning, artificial tooth cleaning)
- Improving the resident’s oral function
- Improving dining-assistance skills
- Reviewing the dining environment
- Improving the resident’s general health condition
- Regulating the resident’s life rhythm

Items were jointly selected with residents and staff members after displaying them on a whiteboard to increase visibility. The researchers and staff members held additional discussions about the direction of care when difficult cases were encountered during the intensive intervention. This ensured that each care program respected individual needs and preferences.

### Part 2: Three-month Program Continuation Period

A survey was conducted 3 months after beginning the intensive intervention. Based on this survey, strengthening items were chosen from the list above that would be implemented during the next 3 months. This also involved discussions with residents and staff members. All planned activities were written on the whiteboard.

### Participants and Ethical Considerations

A nonprobability convenience sampling technique was used to recruit participants from three senior welfare facilities in Japan. Specifically, this included elderly residents (60 years of age and older) and staff members who provided them with care. All participants were given oral and written explanations of this study’s procedures. Further, written consent was obtained prior to their participation. However, some residents had difficulty making the decision to participate by themselves. The researchers not only attempted to directly obtain consent in these cases but also accepted written proxy consent from their families when necessary. Approval to conduct research involving human subjects was received from the research ethics committee of the College of Nursing Art and Science, University of Hyogo (KYOUIN24) on March 12, 2013.

### Research Items

#### Care records for process evaluation

We asked staff members to record information about the type of care they provided to residents based on what they derived during the learned sessions. Staff members were interviewed to assess this information at three time points (1 month, 2 months, and 3 months after the intensive intervention began).

### Demographic Data

Residents were surveyed to collect demographic information, including gender, age, medical history, and care levels. Based on standards established by the MHLW, care needs were classified into five levels (1 = lightest, 5 = heaviest) (JMHLW, 2013). An individual with a care level of l required assistance in some daily living activities due to reduced muscular strength, while an individual with a care level 5 was bedridden and/or unable to eat without assistance, thus requiring constant care for all daily living activities.

### Measures

We collected data at four points, including the beginning of the program (baseline), 3 months after the program began (month 3), the end of the program (month 6), and 6 months after the end of the program (month 12) (Figure 2).

#### 1. Nutritional Status

Body mass index (BMI) measurements were determined for each participant using their heights (m) and bodyweights (kg) ([body weight]/[height]2). This was used as an index for nutritional status.

Each participant was also assessed for daily caloric intake by multiplying the daily calorie contents of their meals by the rate of intake (reflected by weight). Mean daily calorie intake was thus determined for each of the three days just prior to measurement date. Finally, daily water intake was determined using records from the nursing station. Mean daily water intake was thus calculated for each of the 3 days just prior to measurement date.

#### 2. Oral Conditions

A dentist examined each participant to determine oral condition, including dental plaque (Greene & Vermillion, 1960), tongue coating (Miyazaki, Sakao, Katoh, & Takehara, 1995), and tongue movement (Sakashita et al., 2016). This was done with careful consideration of the lighting conditions while following the criteria shown in Table 1.

**Table 1:** Assessment Scores for Oral Condition and Tongue Movement

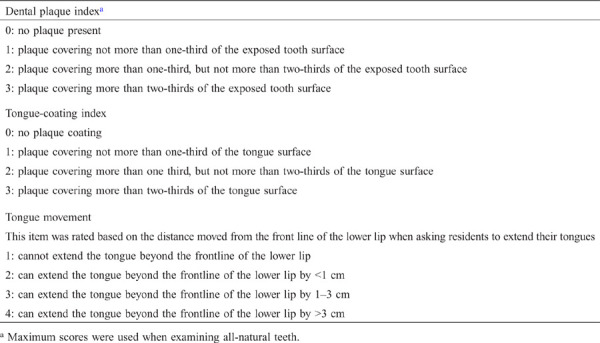

#### 3. Vitality and Appetite

The vitality index (VI) was developed to assess QOL for older people with dementia (Toba et al., 2002). Vitality was specifically expressed on a scale ranging from 0 to 10, with higher scores indicating higher levels of willingness.

Participants also rated their appetites by selecting one of four options, including 1 = no appetite, 2 = uneven appetite, 3 = moderate appetite, and 4 = good appetite.

#### 4. Frequency of Fever during the Previous 3 Months

Resident records were checked for the presence/absence of fever (body temperatures exceeding 37.5°C or a ≥ 1°C increase over an individual’s average temperature) and frequency was counted during the previous 3 months before measurement. Body temperatures were measured at fixed times each day, with median temperatures from the previous week (seven-day period) used as averages for each resident.

### Data Analysis Methods

As mentioned above, quantitative data were obtained at four time periods for the purpose of comparison. Variables with normal distributions based on the Kolmogorov–Smirnov test were subjected to the repeated measures ANOVA test, while variables with nonparametric distributions were subjected to the Friedman test. All statistically significant differences were subjected to multiple comparisons via the Bonferroni post hoc test. Here, statistical significance was set to *P* < 0.05. All statistical analyses were conducted using PASW Statistics v17 (SPSS Inc., Chicago, IL, USA). Finally, interventional programs were qualitatively assessed based on individual care records.

## Results

### Participant Characteristics

Data were obtained and analyzed from 168 residents (31 males and 137 females; mean ± S.D. age of 85.9 ± 8.2 years) from three senior welfare facilities (Table 2). Data obtained from different genders and the three welfare facilities were integrated for further analysis due to the limited marked differences in subject composition and research results. All residents suffered from some type of disease (*e.g.*, 136 [81.0%] were diagnosed with dementia, 95 [56.5%] had hypertension, and 83 [49.4%] had cerebrovascular disease) (Table 2). Participants also included 151 staff members, including 125 care works, 7 nurses, 16 therapists, and 3 dieticians.

**Table 2:** Participant Baseline Characteristics (Residents)

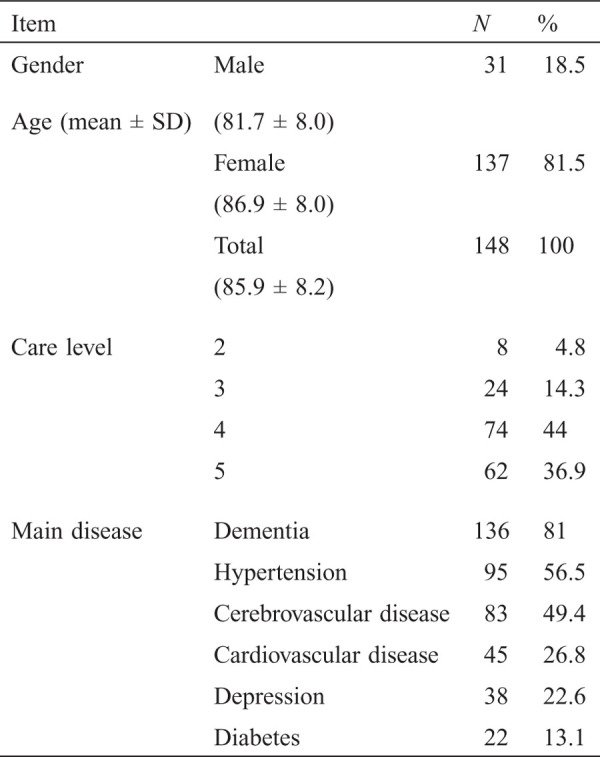

### Care Provided through the Program

Baseline data showed that 87 (51.8%) residents had insufficient chewing/swallowing functions for eating solid foods, while all residents had substantial dental and/or tongue plaque. The first intervention session therefore included components designed to improve oral function and hygiene. Care records showed that staff members responded by providing residents with oral function care (*e.g.*, salivary-gland stimulation massage, tongue massage, and oral motor exercises) and oral hygiene care (based on individual needs).

Care records showed that staff members started to provide oral care at the first, then gradually made efforts to enhance the dining experience. Specifically, this included arrangements designed to improve comfort levels in the dining areas, place-setting the dining tables in appealing ways, and creating enjoyable atmospheres. Further, records showed that various types of related care were provided (*e.g.*, setting the tables in appealing ways and creating enjoyable atmospheres). After the third session, care records showed that staff members began to review and regulate life rhythms, improving more general conditions for residents. This included assisting residents when getting out of bed, ambulating with self-propelled wheelchairs, increasing opportunities for walking, creating comfortable bedroom environments to promote sleep, and providing support to ensure better excretion control.

### Program Effects (Table 3)

#### Nutritional Status

Caloric intake had increased significantly by the end of the program (month 6) when compared to baseline measurements, but returned to preintervention levels when measured 6 months after the program ended (month 12). Further, water intake significantly increased by months 3 and 6 when compared to baseline measurements but decreased by 6 months after the program ended (month 12) when compared to the level of month 3. Finally, BMI had significantly increased by month 3 when compared to baseline measurements but returned to preintervention levels by 6 months after the program ended (month 12).

**Table 3:** Effects of the Dining-focused Life Enhancement Program

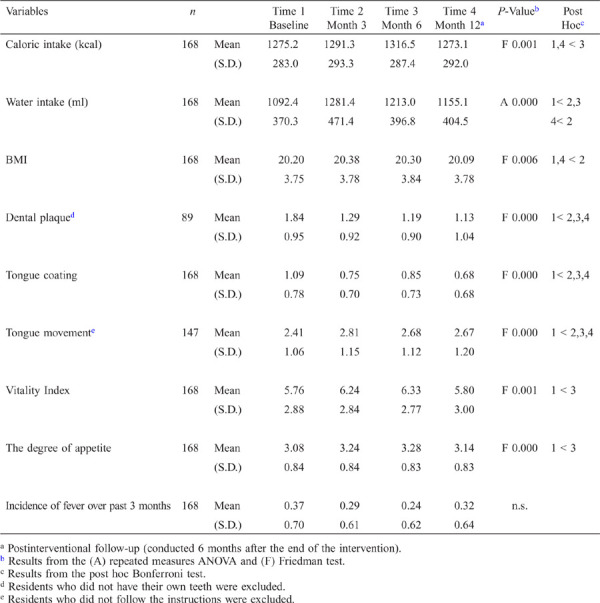

#### Oral Health

Postintervention results showed significant reductions in dental plaque, tongue coating, and tongue movement.

#### VI and the Degree of Appetite

While postintervention VI and the degree of appetite indicators significantly increased from baseline, residents tended to return to preintervention levels based on final follow-ups.

#### Fever Frequency

No significant changes were observed for fever frequency.

## Discussion

### Resident Characteristics

Resident demographic data for age, gender, and care needs were very similar to those revealed for elderly subjects based on a national survey in Japan (JMHLW, 2016). In both datasets, most individuals were over 80 years of age, with high proportions of women. However, only 81% of the residents in this study had been diagnosed with dementia, compared with 98% in the national survey. This difference is likely because the national survey did not determine dementia based on medical diagnoses, but established the condition based on criteria related to independence in the context of daily life. As such, the residents in this study were deemed suitable for addressing the research questions.

### Program Effects

Results showed that the program significantly improved nutritional status, oral health, appetite, and VI scores among residents. However, fever frequency did not change.

Oral health care improvements were proposed for the intensive program since all residents exhibited problems in this area. Such improvements also promoted dining habits and conditions by increasing their ability to eat. This indicates that oral function can be improved and maintained by keeping the oral environment clean. Further, our previous study found that oral hygiene was easy to implement since residents and caregivers noticed the issue (Sakashita et al., 2016). For that reason, oral hygiene was maintained even after the intervention ended.

During the second proposal session for the intensive program, researchers suggested that staff members review the dining environment and devise ways to help residents to enjoy their dining experiences. Yamada’s (2003) interventional study among 16 elderly dementia patients with eating difficulties found that two components were essential for feeding support, including “environmental arrangements for drawing out self-care ability” and “physical and social environmental arrangements to facilitate adaptation.” Research has also found that individuals with declined cognition require dining environments that allow them to concentrate on eating while keeping them more interested in their meals (Mamhidir, Karlsson, Norberg, & Mona, 2007). This study thus ensured that various care activities were implemented to promote a comfortable dining environment (Figure 1). Results showed that this improved appetites and caloric/water intake, thus leading to BMI gains.

During the third proposal session, researchers suggested that staff members attempt to regulate life rhythms and improve general health conditions for residents. Care activities were thus extended to all aspects of life. This appeared to contribute to the improvements seen in VI scores, which are used to assess QOL for older persons with dementia and related to life prognoses (Toba et al., 2002).

As mentioned above, however, there were no significant effects on fever frequency. This is likely because there were already very few incidents both at baseline and during intervention.

### Care Based on Interrelationships

Professional caregivers tend to focus on routine tasks and safety rather than fulfilling individual needs and preferences (Martha, 1970). In such cases, residents may lose their independence. For instance, they may have no choice in their mealtimes and contents. This makes it more likely that residents will experience reduced appetites and thus consume less food (Groher & McKaig, 1995). For that reason, it is necessary for caregivers to build relationships with residents that entail respect and dignity, thus eliciting autonomy when deciding on things such as meals. In this regard, it is also recommended that caregivers work with the families of residents to help them discover their roles while creating opportunities to fulfill them (Keller et al., 2018). There are also smaller issues to consider. For example, research has shown that interpersonal relationships can be enhanced by using the person’s name or nickname when engaging with them (Watkins, Goodwin, Abbott, Hall, & Tarrant, 2017). Such interactions corresponded to the antecedent factors of “appetite/desire to eat” and “persons to meal with.”

In this study, interrelationship-based care training was considered effective for teaching staff about the importance of recognizing residents as “dinner guests” while promoting person-centered care rather than approaching the issue solely from the caregiver perspective. These types of positive interactions between residents and staff members may increase vitality among residents. This also includes making efforts to share information. For instance, residents and staff members may use a whiteboard to write jointly decided goals.

### Continuance of Effects in the Postprogram Setting

Excluding oral health, the interventional effects tended to decrease based on 6-month postinterventional follow-up results. While several previous interventional studies have examined dining assistance (Athlin, Norberg, & Asplund, 1990; Simmons et al., 2008), few of these investigated whether the interventional effects remained in the poststudy setting. Since residents living at welfare facilities require high levels of care, it is difficult for them to maintain good health without support from staff members. This makes it highly important to ensure that care provisions are continually implemented. Further, caregivers need special skills to care for residents who have various health problems, including dementia and vulnerability (Bradshaw, Playford, & Riazi, 2012). Reports have shown that caregivers may not only cause physical fatigue but also present significant psychological burdens (Sun, Mainland, Ornstein, Sin, & Herrmann, 2018). In this regard, staff members need to ensure stability by providing continual and appropriate support.

This study’s effects on oral cleaning were maintained postintervention, which was similar to the results of a previous study targeting community-dwelling elderly persons (Sakashita, Hamada, Sato, Abiko, & Takami, 2017). This is likely because oral health status is easy to visualize and demonstrate. Further, individuals receiving oral health interventions can directly feel the effects of continual efforts designed to improve hygiene (Sakashita et al., 2017).

### Study Limitations

Since there is no control group for the study and randomization, this study has limitations to conclude a causal association between interventions and outcomes.

The program implemented in this study was not focused on treating individuals in the terminal phase. As such, future studies should develop tailor-made programs to address this issue. There was also a tendency for the interventional effects to decrease postintervention. It is thus necessary for future studies to consider additional measures and strategies that can help residents maintain positive interventional effects.

## Conclusion

This study implemented a comprehensive life-enhancement program that focused on dining conditions for residents of a senior welfare facility in Japan. The program produced notable improvements to their oral health, interest in food, vitality, and nutritional status, but did not have any significant effects on fever frequency. Excluding the persistent improvements to oral health, however, these positive effects tended to disappear postintervention. This indicates the importance of ensuring that welfare facilities provide residents with continual daily support for these matters. This especially includes those with decreased functional abilities, who require additional support when attempting to maintain good health and proper nutrition.

## Acknowledgments

We would like to thank the following people for their cooperation and support during this study: nursing care station residents and their families, staff members, and study collaborators.

## Declaration of Conflicting Interests

The authors declared no potential conflicts of interest concerning the research, authorship, or publication of this article.

## Funding

This research was funded by a Grant-in-Aid for Scientific Research between 2012 and 2014 (Grant-in-Aid for Scientific Research (B)) (Japan Society for the Promotion of Science, KAKENHI, Grant Number JP24390511, PI: Reiko Sakashita).
